# Pyrolyzed Agro-Food By-Products: A Sustainable Alternative to Coal

**DOI:** 10.3390/ma18071495

**Published:** 2025-03-27

**Authors:** Lukáš Jeníček, Jan Malaťák, Jan Velebil, Michal Neškudla

**Affiliations:** Faculty of Engineering, Czech University of Life Sciences Prague, Kamýcká 129, 165 00 Prague, Czech Republic; malatak@tf.czu.cz (J.M.); velebil@tf.czu.cz (J.V.); neskudla@tf.czu.cz (M.N.)

**Keywords:** biomass, biochar, calorific value, energy residual, spruce wood, spent coffee ground, tea waste, nut shells

## Abstract

This study investigates the potential use of biochar derived from residues—such as spruce wood, spent coffee grounds, tea waste, and nutshells—as a sustainable coal substitute—to enhance the decarbonization of European energetic systems and decrease the dependence on fossil fuels. The biomasses were pyrolyzed at 250–550 °C, analyzed for calorific value and composition, and evaluated for energy retention and mass loss. The results show significant energy density improvements, with optimal temperatures varying by material (e.g., spruce wood reached 31.56 MJ·kg^−1^ at 550 °C, retaining 21.84% of its mass; spent coffee grounds peaked at 31.26 MJ·kg^−1^ at 350 °C, retaining 37.53%). Economic analysis confirmed pyrolyzed biomass as a cost-effective alternative to coal, especially considering emission allowance costs. Integrating biomass pyrolysis into regional energy systems supports decarbonization, reduces emissions, and advances us towards a circular economy.

## 1. Introduction

The dwindling reserves of fossil fuels, coupled with Europe’s goal to reduce dependency on them [1], have spurred interest in sustainable alternatives like biomass waste within a circular economy framework. Renewable energy sources such as solar, hydro, wind, and biomass are crucial for sustaining economic growth while protecting the environment [2]. Biomasses from agricultural and forestry by-products such as sawdust, bagasse, coffee husk, and nutshells are already commonly used as solid fuels in developing regions [3,4,5] and have the potential to replace fossil fuels in developed countries [6,7]. Biomass can also be co-combusted with coal in existing coal power plants in order to achieve reduced greenhouse gas emissions and reduced solid waste from the combustion process [8]. The results show that blends with 70 wt. %, 50 wt. % and 30 wt. % biomass could reduce the non-renewable CO_2_ emissions per produced unit of energy by about 75%, 57% and 34%, respectively [9]. Purohit and Chaturvedi [10] estimated that associated carbon dioxide mitigation potential in India would be 205 Mt in 2030–2031 if the entire biomass surplus is to be diverted for power generation. Coal remains the primary global fuel for heat and power production, but its use significantly contributes to greenhouse gas emissions [11]. Major CO_2_ emitters include China, the USA, India, and European nations such as Germany and Poland [12]. Finding viable alternatives to fossil fuels remains a pressing challenge [13,14,15]; however, pyrolysis technologies utilizing renewable biological resources show promise in producing substitutes for coal [16].

Recent advancements in biochar research have further reinforced its potential use as a sustainable energy resource. For example, a study from Prabha [17] highlighted that biochar could significantly contribute to improving soil health and mitigating climate change. This is due to its carbon sequestration capabilities and its ability to act as a clean-burning fuel source. Other studies, such as one by Rajput [18], have emphasized the role of optimized pyrolysis processes in tailoring biochar properties to enhance its calorific value and material applications, ranging from renewable energy to industrial uses. These findings underline the critical importance of refining production methods to maximize both efficiency and versatility, paving the way for broader applications across sectors.

A bibliometric analysis of biochar trends published by Wu [19] demonstrated a growing focus on the economic feasibility of biochar applications in agricultural, environmental, and energy sectors. Such advances underscore the importance of improving biochar’s production efficiency and scalability, ensuring that its benefits extend beyond niche uses to become a cornerstone of renewable energy strategies.

In the Czech Republic, energy policy prioritizes the use of alternative biomass resources like damaged timber and logging residues for fuel, aligning with plans to decarbonize the energy sector [20,21]. The region’s significant challenges, including extensive forest disturbances [22,23] caused by climate change [24,25,26,27] and associated salvage logging [28], provide a substantial supply of by-products suitable for biochar production. These by-products not only offer an environmentally friendly solution for waste management, but also present an economically viable resource for renewable energy [29].

One of the key technologies for enhancing biomass energy properties is low-temperature pyrolysis, often called torrefaction, a process involving the slow heating of organic materials like wood, spent coffee ground [30,31,32], straw, and nutshells in a low-oxygen environment. This method not only improves the energy properties of biomass, but also makes it a more effective, clean-burning fuel [33,34,35]. Pyrolysis increases biochar’s energy density, making it easier to handle and transport, and reduces overall logistics costs. Furthermore, biochar production supports renewable energy generation and aids in carbon sequestration, which is crucial for mitigating climate change [36,37].

Recent research has demonstrated that pyrolysis can be optimized using machine learning techniques to enhance energy recovery and adapt material properties to specific applications [38,39]. Studies have also explored the co-pyrolysis of multiple biomass types, revealing synergistic effects that enhance yield and quality, a promising area for further exploration [40,41].

Economic evaluations have also underscored the viability of using biochar as a substitute for coal. The economic and environmental benefits of transitioning to biochar-based fuels are supported by their lower emissions [42], reduced waste disposal costs, and ability to create new markets for agricultural by-products [43,44,45,46]. Studies, including those examining the use of biochar in decentralized energy systems, emphasize the efficiency of localized production and utilization [47,48,49], with additional benefits such as soil conditioning for biochar [50]. Decentralized approaches not only reduce the environmental costs associated with transportation, but also increase resilience by enabling energy generation close to biomass sources. These findings further bolster the argument for incorporating pyrolyzed biomass into the Czech Republic’s energy mix [51], supporting EU decarbonization goals and promoting circular economy approaches.

In summary, advancements in biochar research and its integration into renewable energy policies suggest that optimizing local waste materials for energy production contributes towards achieving energy security and sustainability in the region. This study aims to evaluate the potential of pyrolyzed biofuels made from residues and waste materials, including spruce wood, spent coffee grounds, tea waste, walnut shells, pistachio shells, and peanut shells, as substitutes for coal. Aside from comparing the proximate and ultimate analysis results with those of other authors, this article also focuses on the economic aspect of biochar usage.

By leveraging recent technological and economic insights, the potential for biochar to transform energy systems and contribute to environmental sustainability is made increasingly apparent.

## 2. Materials and Methods

To confirm data consistency, results from prior research conducted in 2020–2022 [52,53,54,55] are here re-evaluated alongside new analyses performed in 2023. Additional analyses were conducted on the samples to assess the overall energy potential of these sustainable biochar materials as substitutes for fossil resources.

The selected pyrolysis temperatures (250 °C, 300 °C, 350 °C, 450 °C, and 550 °C), along with a non-pyrolyzed sample (designated as 0 °C), were chosen to represent the full range of slow pyrolysis processes, from low to moderately high temperatures. Pyrolysis at 250 °C induced notable changes in material composition for most samples, as illustrated in the Van Krevelen diagram (Section 3.5), with a minimal correlation between temperature and mass loss [56]. In contrast, pyrolysis at 550 °C led to highly active pyrolysis reactions, depending on the nature of the tested materials [57], resulting in the transformation of the material into gaseous and liquid states. These selected temperature points align with those from previous studies, ensuring methodological consistency and enabling reliable comparisons across datasets.

The samples, sourced from renewable energy materials, were selected as by-products from the forestry and food industries. The primary focus was to determine the optimal energy utilization of these samples as substitutes for fossil fuels.

Spruce timber was harvested from the Vysočina region in the Czech Republic in 2023. The samples, sourced from logs cut 12 to 18 months earlier, were processed into wood chips and combined to form a representative composite sample. In 2020, the EU had around 159 million hectares of forests, a 10% increase since 1990. Forest areas grew in most EU countries, with Ireland, Spain, and Malta experiencing notable expansions. In 2019, the EU’s wood stocks were 28.4 billion m^3^, with Germany, Sweden, and France leading in volume [29]. The Czech Republic’s wood volume rose by 15%, representing 2.7% of European wood stocks [58].

Spent coffee grounds (SCG) were obtained from *Coffea arabica*, which constitutes approximately 75% of global coffee production [59,60]. These grounds, collected from a single household (4 kg total), were sun-dried for several days until reaching a stable moisture content of 8.45%. Coffee, as one of the world’s most widely consumed beverages, generates substantial amounts of spent coffee grounds, totaling approximately 6.5 megatons annually worldwide [61]. In the Czech Republic, about 3 billion cups of coffee consumed in 2019 produced roughly 24,000 tons of SCG [62]. Rich in cellulose, sugars, minerals, and lipids, coffee beans are valuable for producing biodiesel or fuel pellets, with a calorific value of about 20.9 MJ·kg^−1^ [63,64,65].

Black tea residues (*Camellia sinensis* L. Kuntze) were gathered from domestic brewing processes over four months and sun-dried to reach a moisture content of 8.31%. Tea is the second most popular non-alcoholic drink globally, with nearly 6 million tons produced annually [66,67,68]. In Czech Republic, the annual production is approximately 2000 tons of tea waste [62]. Like coffee, tea consumption generates significant waste, with over 90% of the tea material left unused after brewing [69]. Studies have demonstrated tea waste’s potential as a fuel pellet resource, showing high calorific values and suitability as a natural additive to other biomass materials [70,71,72,73,74]. However, tea waste-based pellets may contain high ash levels and deposit-forming elements [75,76].

Nutshells, namely, walnut shells harvested from English walnut (*Juglans regia* L.), pistachio shells from *Pistacia vera* L., and peanut shells derived from *Arachis hypogaea* L., were also used. The United States is a leading producer of nuts [77,78], with its industries generating significant by-products. In California, there were 1.5 million tons of almonds, 0.5 million tons of pistachios and 0.4 million tons of walnuts produced [79], while in Czech Republic, 22 tons of nut of waste was generated [62] in 2020. Walnut and almond shells, often discarded, can be converted into biochar, offering enhanced properties and reduced emissions compared to direct combustion. Processing companies report that utilizing nutshells as biochar or biofuel offsets disposal costs and provides an environmentally friendly alternative [80,81,82,83,84,85,86].

All samples were ground to a particle size of 1 mm using a Retsch SM 100 mill (Haan, Germany) and then subjected to pyrolysis in a LECO TGA 701 analyzer (St. Joseph, MI, USA) at five distinct temperatures: 250 °C, 300 °C, 350 °C, 450 °C, and 550 °C. Each sample was heated in an inert atmosphere at a rate of 10 °C per minute and held at the target temperature for an additional 30 min. The 30-min residence time was selected based on the available literature, as this duration has been shown to optimize pyrolysis performance. This has been demonstrated in the processing of fruit bio-waste [87], as well as in the pyrolysis of olive pits [88].

### 2.1. Proximate and Ultimate Analysis

Each sample underwent proximate and ultimate analysis to understand its material composition and characteristics. In the proximate analysis, measurements included elemental composition, moisture content, and ash content. The ultimate analysis involved determining the calorific value [89]. All measurements were conducted 5 times, and the average value was used for the following calculations.

A LECO TGA 701 thermogravimetric analyzer was used for measuring moisture and ash contents [90]. To calculate moisture content, 1 g of each sample was dried at 105 °C to a constant weight [91]. For ash content, 1 g of each sample was heated to a constant weight up to 550 °C.

The chemical compositions were analyzed using a LECO CHN628+S elemental analyzer (LECO Corporation, St. Joseph, MI, USA) through the LECO instrumental biomass combustion method. Each 0.1-gram sample was burned in oxygen at 950 °C to measure the amounts of carbon (C), hydrogen (H), and nitrogen (N). The oxygen content was determined by difference to a total of 100% on a dry basis. The LECO elemental analyzer was regularly calibrated using ethylenediaminetetraacetic acid, rice flour, and oatmeal.

To determine the calorific value, 1 g of each sample was placed in a stainless-steel crucible and secured in a cylinder. The pressure increased to 3 MPa at a reference temperature of 28 °C. The sample was then ignited with a cotton thread inside a LECO AC600 isoperbolic calorimeter (LECO Corporation, St. Joseph, MI, USA). Benzoic acid was used to calibrate the instrument. All proximate and ultimate analysis results were reported on a dry basis.

Net calorific value was calculated from the measured gross calorific value using the equation

(1) $$Q_{n}=Q_{v}-(24.42\times (\left(W\right)+\left(8.94\times H\right))$$

where Q_n_ (kJ·kg^−1^) is net calorific value, Q_v_ (kJ·kg^−1^) is gross calorific value, W (%) is water contain in the sample, H (kg·kg^−1^) is hydrogen content in the sample, 24.42 is the amount equal to energy needed to evaporate 1% of water at 25 °C and 8.94 is the coefficient for the conversion of hydrogen to water.

### 2.2. Energy Residual

The energy residual of the original mass was calculated by adjusting the calorific value for the material’s enthalpy and accounting for the mass loss.

The enthalpy of the material (kJ·kg^−1^) was determined using the standard for spruce wood. Weight loss was measured with a LECO TGA 701 (LECO Corporation, St. Joseph, MI, USA). During the material’s pyrolysis, weight measurements were taken with an accuracy of 10^−4^ g.

### 2.3. Thermogravimetric Analysis

To express the dynamics of volatile matter loss during heating, LECO TGA 701 was used to perform a TGA analysis. The samples were enclosed in aluminum foil and put in covered crucibles. The temperature program consisted of two phases. In the first one, the samples were dried at 105 °C until constant weight. In the second, the temperature would ramp up at a rate of 5 °C min^−1^ from 105 °C to 580 °C. The results were processed into weight loss curves (TGA) and their derivations (DTG curves). The values were converted to express the loss of combustible matter. The DTG curves were then taken to represent the rate of loss (wt. % min^−1^) of combustible matter as a function of temperature.

### 2.4. Economic Assessment of Material Pyrolysis

An economic assessment was performed considering the price of the material and energy residual from the pellet produced.

Pellet production was calculated as a fixed energy cost E_p_.

(2) $$Q_{P}=Q_{T}-E_{p}$$

where Q_P_ (kJ·kg^−1^) is energy residual in the pellet, Q_T_ (kJ·kg^−1^) is energy residual in the material and E_P_ = 108 kJ·kg^−1^ is the fixed energy cost of pellet production [92].

The final price of 1 MWh (EUR) for all materials in the form of a pellet was calculated by use of the following equation:

(3) $$P_{MWh}=\frac{P_{kg}}{Q_{P}*0.2778}\times 1000$$

where P_MWh_ (EUR) is a price of 1 MWh, P_kg_ is a price of 1 kg, Q_P_ (kJ·kg^−1^) is energy residual in the pellet and 0.2778 is a coefficient to display kg in MWh.

### 2.5. Statistical Analysis

The statistical analysis of the results was conducted using the STATISTICA 14.0.0.15 CZ (StatSoft, Tulsa, OK, USA) software. The data were subjected to a one-way ANOVA.

## 3. Results and Discussion

### 3.1. Proximate and Ultimate Analysis

Elemental composition, ash and water content, and calorific value were measured for all samples. Measurements were performed five times for all the samples. The final average results with the given deviation are shown in the Table 1.

The overall results of the proximate and ultimate analysis of the present materials do not fundamentally differ from those reported by other authors, e.g., from the processing of olive pits [88] and other agricultural waste materials [87]. The only notable differences are in moisture and ash content, which are specific to the region where the original samples were collected and have a significant impact on the net calorific value.

The spruce wood samples’ results are very close to those of Jenicek et al., Malatak et al. and Arias et al. [53,93,94].

The spent coffee ground proximate analysis results are in line with those of Fermoso and Mašek, Masek et al. and Mayson and Williams [95,96,97]. The calorific values of SCG are similar to those found by Silva et al. and Colantoni et al. [98,99].

The tea waste results here are similar to those found by Tunklova et al. [54], while the calorific value in the non-torrefied sample here is bit lower than that in Sermyagina et al. [100], whose result was 20.39 MJ·kg^−1^.

The results for nutshells are very close to those found by Jenicek et al. and Vassilev et al. [55,101] in all aspects of proximate and ultimate analysis.

### 3.2. Calorific Value and Energy Residual of the Samples

The net calorific value in the dry state (Table 2) was calculated from the calorific value, which was measured using the LECO AC600 isoperbolic calorimeter (Table 1).

The results here are very close to those of Malatak et al. [93], Gendek et al. [102] and Jenicek et al. [42] for spruce wood, with 18.6 MJ·kg^−1^ for dried material and 31.56 MJ·kg^−1^ for the sample pyrolyzed at 550 °C.

Colantoni et al. [99] and Silva et al. [98] reported the calorific values of spent coffee grounds with approximate results of 20.92 MJ·kg^−1^ and 22.36 MJ·kg^−1^, respectively. Similar results were also achieved by Kovalcik et al., McNutt and He, Nepal et al., Bejenari et al. and Ballesteros et al. [103,104,105,106,107].

Tea waste net calorific values were close to the results derived by Tunklova et al. [54], in the range of 17.04–24.31 MJ·kg^−1^ depending on the level of pyrolysis, but were slightly lower compared to Sermyagina et al. [108], who reported gross calorific values of 20.39 MJ·kg^−1^ in green tea and 20.26 MJ·kg^−1^ in black tea.

Measuring the nutshell’s net calorific value, we find results close to those of Jenicek et al. [55], who used the same method, with a walnut net calorific value of 18.66 MJ·kg^−1^ (non-pyrolyzed) to 30.55 MJ·kg^−1^ (550 °C), a pistachio net calorific value of 17.58 MJ·kg^−1^ (non-pyrolyzed) to 31.35 MJ·kg^−1^ (550 °C) and a peanut net calorific value of 19.93 MJ·kg^−1^ (non-pyrolyzed) to 28.78 MJ·kg^−1^ (550 °C).

Material enthalpy for spruce wood was applied for all samples to estimate the energy needed for material pyrolysis. Enthalpy varied from 0.274 MJ·kg^−1^ for the material pyrolyzed at 250 °C to 0.602 MJ·kg^−1^ at 550 °C. The results for net calorific value adjusted by material enthalpy are shown in Table 3.

Another important parameter when assessing the pyrolyzed material is the weight loss under each process temperature. There is an overview in Table 4 of what percentage of original mass remained after the material was pyrolyzed. The highest level of material loss when pyrolyzed at 250 °C was seen in pistachio shells, with only 72.35% of original mass left. The highest loss at 550 °C occurred for spent coffee grounds, which kept only 20.05% of their original mass.

When combining the net calorific value of the pyrolyzed material adjusted by the material enthalpy and weight loss of the pyrolyzed material, we get the energy residual of the original mass weight unit. As we can see in Table 5, for spruce wood, pyrolysis led to a decrease in energy residual from 18.600 MJ·kg^−1^ to 6.762 MJ·kg^−1^. In other words, the direct combustion of 1 kg of dried spruce wood would lead to the generation of 18.600 MJ·kg^−1^, or if the same 1 kg of dried spruce wood is first pyrolyzed at 550 °C, the total output would lead to the generation of only 6.762 MJ·kg^−1^. This energy loss was significantly greater than that measured by Liu et al. [109], who showed bamboo pellet mass losses of 2.12%, 3.31% and 7.45% at 180 °C, 200 °C and 220 °C, respectively. The difference in mass loss is caused by the lower temperature in the experiments of Liu, as well as different materials and different processes of pyrolysis.

To summarize the results, Table 2 shows the net calorific value of the sample; the higher pyrolysis of the material leads to higher energy density and therefore higher net calorific value. On contrary, Table 5 shows that the total energy output of the original dried material was lowered by the material enthalpy and the weight loss caused by the pyrolysis. An equilibrium between increasing the material energy density and losing the energy of original mass when modifying the material by pyrolysis needs to be set for each material separately.

### 3.3. Calorific Value Density and Weight Loss Comparison

**Figure 1:**
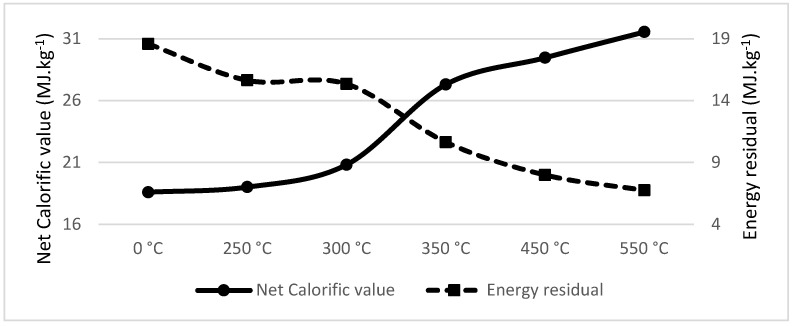
Net calorific value (MJ·kg^−1^) and energy residual (MJ·kg^−1^) of Spruce wood.

Spruce wood showed a significant drop in energy residual between pyrolysis temperatures of 300 °C and 350 °C; in the same interval, spruce wood increases its calorific value density from 20.83 MJ·kg^−1^ to 27.31 MJ·kg^−1^ (see Figure 1). The recommended temperature for spruce wood pyrolysis is 300 °C. This is lower in comparison to that found by Jenicek et al. [53], recommending pyrolysis at 350 °C, as the mass loss was not considered in this respective article.

**Figure 2:**
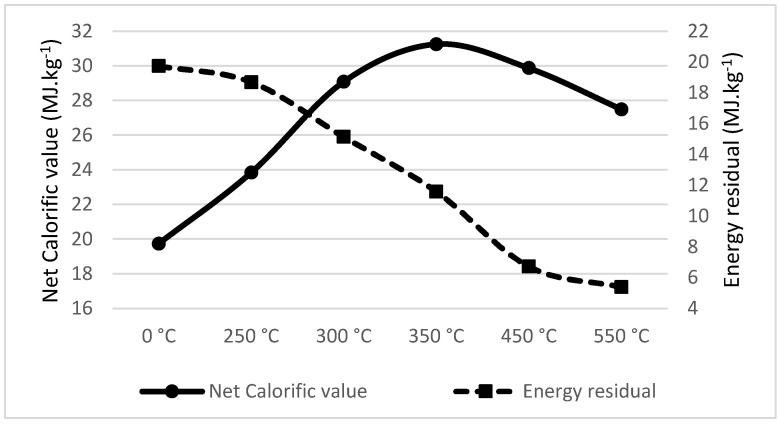
Net calorific value (MJ·kg^−1^) and energy residual (MJ·kg^−1^) of spent coffee ground.

Spent coffee ground dropped in total energy considerably from the temperature level of 250 °C, as seen in Figure 2. The energy density increased substantially between 250 °C and 300 °C. The recommended pyrolysis temperature was also 300 °C, as it was for spruce wood, which is slightly lower than recommended by Jenicek et al. [52], who recommended 350 °C as an ideal pyrolysis temperature for spent coffee ground, and did not show the mass loss figure estimations in their original article.

**Figure 3:**
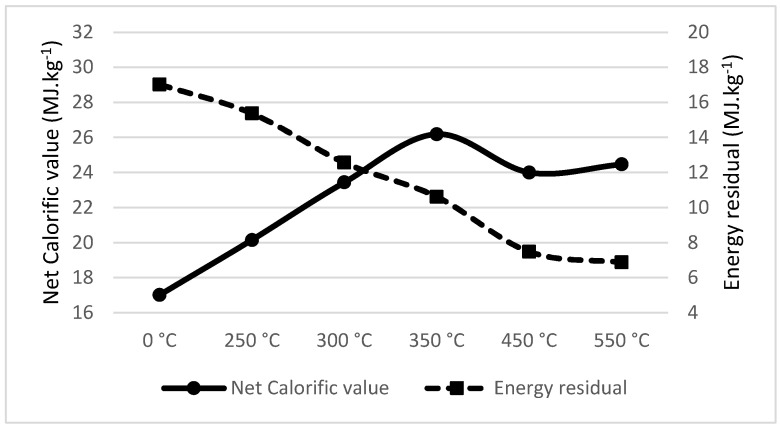
Net calorific value (MJ·kg^−1^) and energy residual (MJ·kg^−1^) of Tea waste.

Tea waste pyrolysis showed a significant drop in total energy produced above the temperature of 250 °C. From the research of Tunklova et al. [54], it can be confirmed that increasing the share of ash also occurred for tea waste pyrolysis; therefore, its recommended temperature is no more than 250 °C. Detailed curve of net calorific values and energy residuals of the pyrolyzed material are visible on Figure 3.

**Figure 4:**
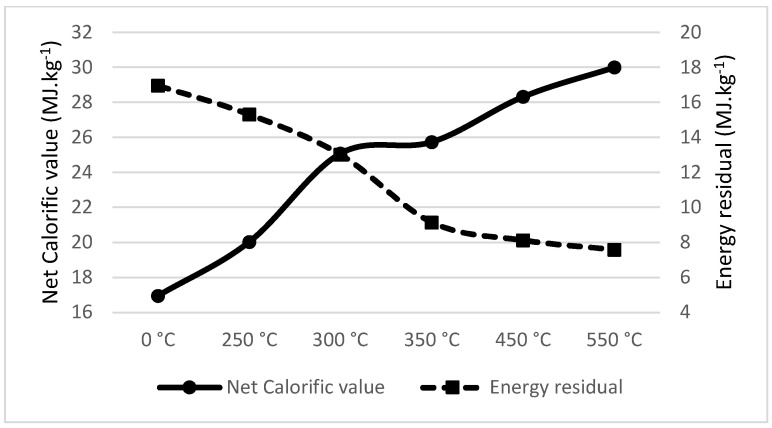
Net calorific value (MJ·kg^−1^) and energy residual (MJ·kg^−1^) of Walnut shell.

Walnut shell pyrolysis significantly increases the net calorific value at the temperature of 300 °C; for higher temperatures, the net calorific value remains stable or increases indistinctly. On the other hand, the energy residual drops at the temperature of 350 °C, as seen in Figure 4. As such, the recommended temperature for the pyrolysis of walnut shell is 300 °C, the same as measured by Jenicek et al. [55].

**Figure 5:**
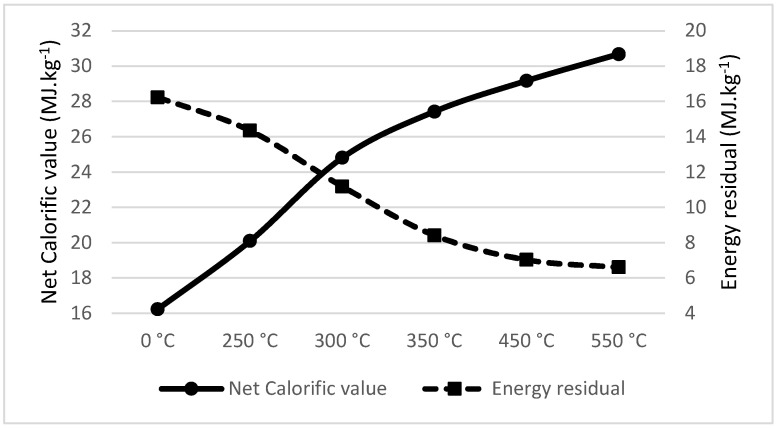
Net calorific value (MJ·kg^−1^) and energy residual (MJ·kg^−1^) of Pistachio shell.

Pistachio shell shows an almost linear increase in net calorific value and an almost linear decrease in energy residual with pyrolysis temperature increases. To keep at least 14 MJ·kg^−1^ of total residual, the recommended temperature for pyrolysis should not surpass 250 °C. This is slightly lower than Jenicek et al.’s [55] recommendation of 300 °C pyrolysis. Detailed results are shown in Figure 5.

**Figure 6:**
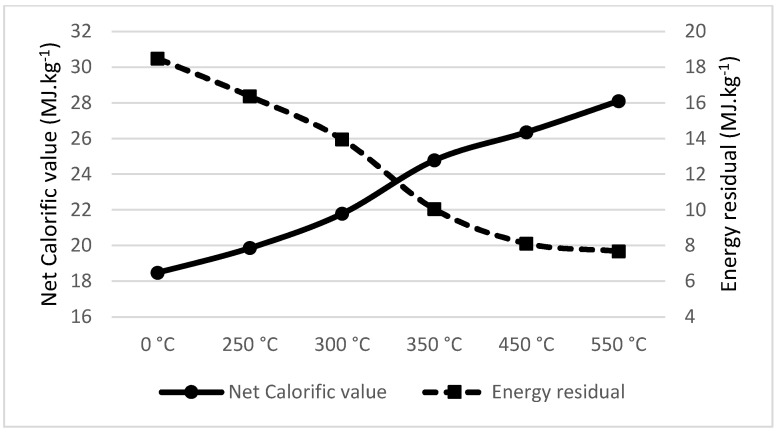
Net calorific value (MJ·kg^−1^) and energy residual (MJ·kg^−1^) of Peanut shell.

Peanut shell shows a similar nearly linear increase in net calorific value and decrease in energy residual with pyrolysis temperature increases. On the contrary, the maximum net calorific value of a peanut shell sample pyrolyzed at 550 °C was 28.11 MJ·kg^−1^ with the energy residual 7.661 MJ·kg^−1^; meanwhile, the pistachio shell sample showed a higher residual net calorific value of 30.68 MJ·kg^−1^ with a lower energy residual of 6.589 MJ·kg^−1^ in the same laboratory conditions. Detailed results are shown in Figure 6. The recommended temperature for the pyrolysis of peanut shell is 300 °C—far lower than suggested by Jenicek et al., who recommended 550 °C.

### 3.4. Economic Assessment of Material Pyrolysis

In order to assess the economic aspects of pyrolysis, let us first adjust the energy residual of dried material by pellet production losses. Pyrolyzed material is pressed into a form of a pellet of 8 mm in diameter and 15 to 20 mm in length. During the pellet production, approximately 0.108 MJ·kg^−1^ [92] of energy is used. The net calorific value of a pellet shown in original mass weight units is presented in Table 6.

In real operation, the production of pellets from material pyrolyzed at high temperatures (more than 400 °C) requires additional components to ensure sufficiently high mechanical durability. This fact is omitted for the purpose of the purity of results of this research [110].

The energy residual of original material combined with the price of the original material gives us the result of price per MWh for each analyzed sample. The spruce wood price was set as EUR 50 m^−3^, which is ca. EUR 106 ton^−1^ of dried spruce wood. The prices of all other materials are very complicated to estimate, as the market is not centralized and not yet well developed; therefore, the prices for spent coffee grounds, tea waste and all nutshells were set as EUR 200 ton^−1^.

The final material prices are shown in Table 7. The lowest price was derived for naturally dried spruce wood, with EUR 20.71 MWh^−1^; the highest price is for spent coffee ground pyrolyzed at 550 °C, with EUR 134.06 MWh^−1^. Recommended pyrolysis conditions are marked in bold letters, with the lowest value of EUR 25.07 MWh^−1^ for spruce wood pyrolyzed at 300 °C, and the highest value of EUR 55.55 MWh^−1^ for walnut shell pyrolyzed at 300 °C.

For a complete overview, these results need to be compared to those of the standard fossil fuel brown coal, which is today still the most widely used fuel around the world. The average price of brown coal for the last 12 months was EUR 110 ton^−1^, which is approximately EUR 28.5 MWh^−1^. Next to the price of coal itself, the price of emission allowances needs to be combined with the 12-month average of EUR 57.58 t(CO_2_)^−1^, which is approximately ERU 86.77 MWh^−1^. In total, this gives over EUR 115 MWh^−1^ in material costs when using brown coal. Due to the high price of emission allowance, any pyrolyzed biomass would be cheaper to use, as it is not burdened by emission allowances. The economic assessment of biomass usage as an alternative to brown coal in municipal power plants was also calculated by Malatak et al. [93], with positive results when considering timber as a more profitable fuel than coal.

Economic evaluations highlight biochar’s viability as a coal substitute due to its lower emissions, reduced waste disposal costs, and potential to create new markets for agricultural by-products [54,55,93]. However, the lack of centralized markets for materials like spent coffee grounds, tea waste, and nutshells leads to price variability. Additionally, collection and transportation costs can significantly impact economic feasibility, particularly for dispersed sources.

Decentralized waste collection, co-locating pyrolysis units near waste generation sites, and integrating with existing supply chains can reduce logistical costs. Future analyses should include transportation impact modeling to improve economic assessments. Strategies such as leveraging local supply chains and minimizing transport distances further enhance cost efficiency.

In summary, while biochar’s benefits are clear, overcoming logistical and economic barriers is vital for large-scale adoption.

### 3.5. Van Krevelen Diagram

**Figure 7:**
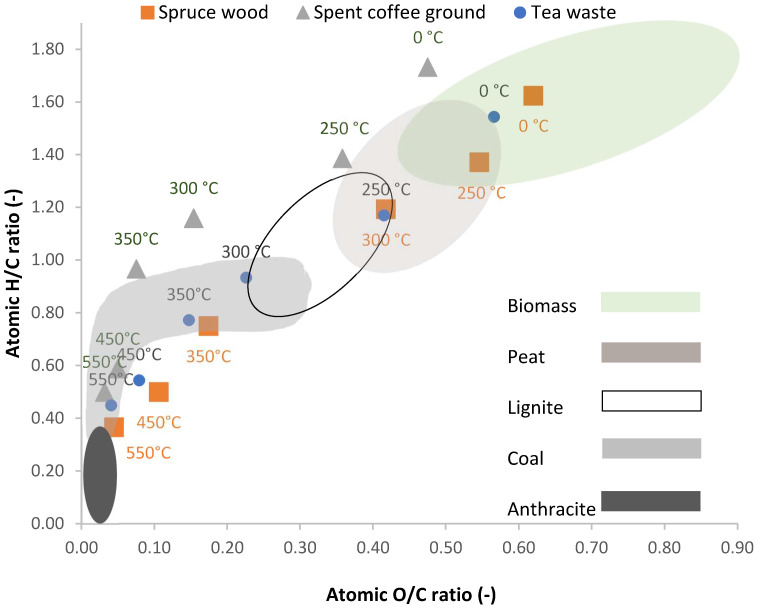
Van Krevelen diagram for spruce wood, spent coffee ground and tea waste ranges for material types according to Van Loo and Koppejan [112].

**Figure 8:**
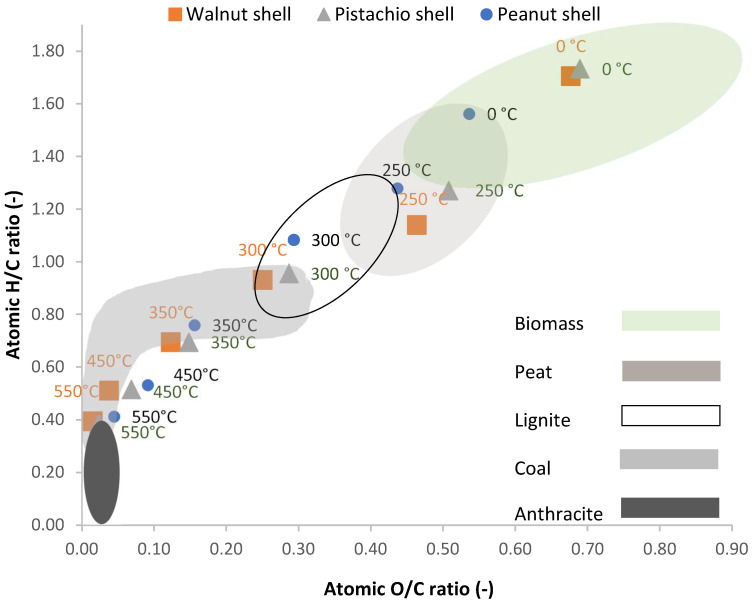
Van Krevelen diagram for walnut shell, pistachio shell and peanut shell ranges for material types according to Van Loo and Koppejan [112].

The van Krevelen diagram (Figure 7 and Figure 8) shows the evolution of the three main components of fuel—carbon, oxygen, and hydrogen. During carbonization, oxygen is released approximately twice as fast as hydrogen until black coal is formed. Further transformation into anthracite decreases the H/C ratio while keeping the oxygen content low.

Figure 7 shows the van Krevelen diagram for biochar produced from spruce wood, spent coffee grounds, and tea waste, where it is evident that increasing process temperatures lead to a reduction in the H/C and O/C ratios. Figure 8 shows similar trends for biochar derived from walnut, pistachio, and peanut shells.

Like fossil fuels, the O/C ratio drops faster than the H/C ratio. Temperatures between 300 and 350 °C cause the greatest decrease in these ratios. For spruce wood, a 100 °C increase lowers the O/C ratio from 0.42 to 0.17, and a further 100 °C increase drops the H/C ratio from 0.17 to 0.11. Similar reductions are seen in other wood biomasses, with the H/C ratio dropping from 1.37 to 0.75 between 250 and 350 °C [111]. Spent coffee grounds and tea waste show ratio changes starting at 250 °C. Walnut, pistachio, and peanut shells display almost identical changes in ratios with rising temperatures.

### 3.6. Thermogravimetric Curves

**Figure 9:**
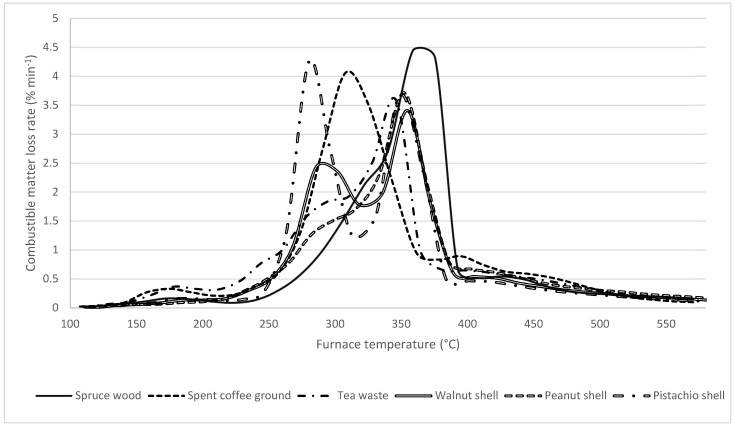
DTG curves of untreated biomass. The curves were converted to express the rate of loss of combustible matter, i.e., dry, ash-free state.

**Figure 10:**
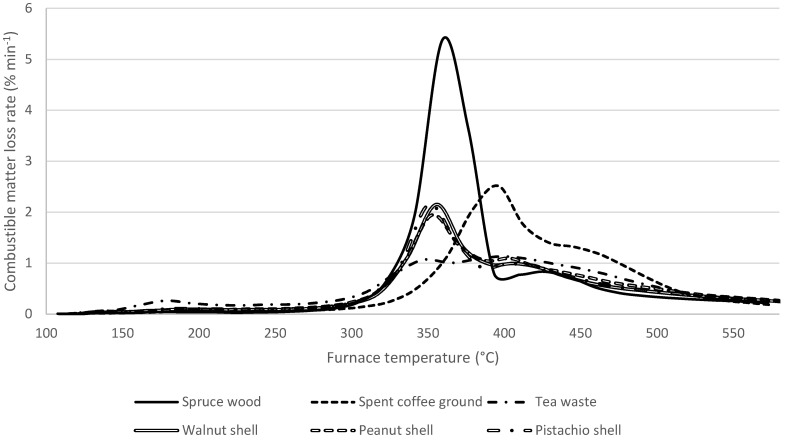
DTG curves of samples treated at 300 °C. The curves were converted to express the rate of loss of combustible matter, i.e., dry, ash-free state.

**Figure 11:**
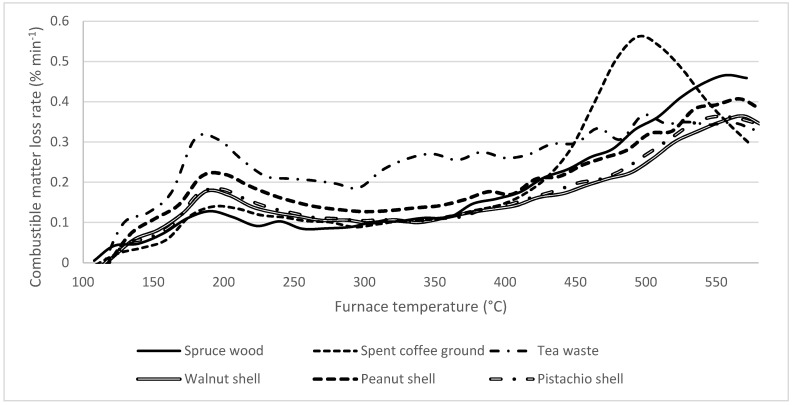
DTG curves of samples treated at 400 °C. The curves were converted to express the rate of loss of combustible matter, i.e., the dry, ash-free state.

To express the differences in the release of volatile matter, e.g., during the first phase of combustion, between the original biomass materials and the biochar, all samples have undergone TGA analysis. The results are expressed as DTG curves of combustible matter in Figure 9, Figure 10 and Figure 11. In raw biomass materials, the spread of results is relatively broad. The significant release of volatiles starts at 250 °C for all materials; however, the trends do vary, owing to the differences in chemical constitution, between the source biomasses, i.e., different proportions of hemicelluloses, cellulose and lignin, all of which tend to decompose in different temperature ranges [113]. In particular, in all samples apart from SCG, the lignin content is apparent in the second DTG peak around 350 °C.

In biochar produced at 300 °C (Figure 10), significant decomposition starts above 300 °C, with the first DTG peak being almost uniform around 350 °C. Markedly different again is the SCG, with a different combustible release profile, possibly thanks to it already being thermally treated as a part of coffee production. Spruce wood biochar achieved a very high maximum (over 5% min^−1^) in volatile release. However, in the other biochar, the release was more gradual, with peaks approximately half as significant compared to raw biomass. This would make the combustion process more similar to coal combustion, or better suited for coal-fired boilers [39,45,48].

In the biochar produced at 400 °C, the DTG curves were much lower than in the previous case, with DTG peaks generally below 0.5% min^−1^. Here, the volatile matter release is not finished by the end of the analysis at 580 °C. In all samples, there is an early peak around 200 °C, which can be inferred to be caused by bound water release. Above this temperature, the profile seems to correlate relatively well with that of, e.g., lignite coal [114,115]. Importantly, the volatile release profiles correlate very well with each other, and therefore are expected to behave similarly in combustion.

## 4. Conclusions

This study underscores the transformative potential of pyrolyzed biomass derived from daily residues and waste materials, such as spruce wood, spent coffee grounds, tea waste, walnut shells, pistachio shells, and peanut shells, as sustainable and high-calorific-value substitutes for coal. By harnessing these underutilized agro-waste resources, which often end up in mixed municipal waste landfills, this approach not only provides a renewable energy solution, but also promotes circular economy principles. These raw materials demonstrate significant promise for local energy applications, ensuring long-term sustainability while maintaining critical quality metrics, such as calorific value and elemental composition. This work reinforces the viability of integrating pyrolyzed biomass into energy systems to reduce dependence on fossil fuels and meet decarbonization goals effectively.

The results demonstrate that pyrolysis significantly enhances the net calorific value of these materials, with optimal temperatures varying based on the feedstock. For instance, spruce wood achieves a net calorific value of 31.56 MJ·kg^−1^ at 550 °C, while spent coffee grounds reach their peak at 31.26 MJ·kg^−1^ at 350 °C. However, higher temperatures also result in greater mass loss, reducing the overall energy retained per unit of original biomass. For example, spruce wood retains only 21.84% of its original mass at 550 °C, while spent coffee grounds retain 37.53% at 350 °C, highlighting a critical trade-off between maximizing energy density and preserving material mass. These findings emphasize the importance of determining material-specific pyrolysis conditions to optimize both energy yield and resource efficiency.

Economically, pyrolyzed biomass offers a cost-effective alternative to fossil fuels when factoring in emission allowances, which significantly increase the cost of coal. The decentralized production of biochar, particularly near waste generation sites, not only reduces logistical and transportation costs, but also supports local economies and waste management strategies. Furthermore, the use of renewable waste materials aligns with circular economy principles, reducing environmental impacts and diversifying energy sources.

In summary, integrating pyrolyzed biofuels into the Czech Republic’s energy framework can play a pivotal role in achieving EU decarbonization targets. By leveraging local biomass resources and prioritizing decentralized energy production, this approach addresses logistical, economic, and environmental challenges, advancing the transition toward a more sustainable and resilient energy system.

## Figures and Tables

**Table 1:** Proximate and ultimate analysis of all samples.

	Carbon (wt. %)	Oxygen (wt. %)	Hydrogen (wt. %)	Nitrogen (wt. %)	Ash (wt. %)	Water (wt. %)	Gross Calorific Value (MJ·kg^−1^)
Spruce wood	47.01 ± 0.82	38.82	6.41 ± 0.14	0.23 ± 0.01	0.33 ± 0.01	7.19 ± 0.09	20.17 ± 0.20
Spruce wood 250 °C	52.00 ± 0.62	37.79	5.98 ± 0.06	0.28 ± 0.01	0.56 ± 0.01	3.38 ± 0.12	20.41 ± 0.24
Spruce wood 300 °C	57.24 ± 0.92	31.84	5.73 ± 0.07	0.33 ± 0.01	0.71 ± 0.01	4.14 ± 0.15	22.18 ± 0.16
Spruce wood 350 °C	72.88 ± 0.61	16.93	4.59 ± 0.03	0.41 ± 0.01	0.92 ± 0.02	4.26 ± 0.05	28.42 ± 0.18
Spruce wood 450 °C	79.00 ± 0.44	11.14	3.32 ± 0.09	0.46 ± 0.01	1.29 ± 0.07	4.78 ± 0.12	30.32 ± 0.17
Spruce wood 550 °C	84.34 ± 0.74	5.03	2.6 ± 0.14	0.56 ± 0.01	1.63 ± 0.07	5.84 ± 0.05	32.27 ± 0.29
Spent coffee ground	49.38 ± 0.82	31.24	7.18 ± 0.05	2.22 ± 0.03	1.63 ± 0.01	8.35 ± 0.11	21.51 ± 0.22
Spent coffee ground 250 °C	59.27 ± 0.84	28.24	6.90 ± 0.04	2.68 ± 0.02	1.99 ± 0.06	0.92 ± 0.01	25.38 ± 0.23
Spent coffee ground 300 °C	70.71 ± 0.67	14.49	6.88 ± 0.11	3.39 ± 0.02	2.96 ± 0.08	1.56 ± 0.02	30.63 ± 0.24
Spent coffee ground 350 °C	76.35 ± 0.66	7.63	6.20 ± 0.06	3.99 ± 0.01	4.10 ± 0.10	1.73 ± 0.03	32.65 ± 0.28
Spent coffee ground 450 °C	77.52 ± 0.84	5.13	3.83 ± 0.10	4.43 ± 0.04	6.38 ± 0.08	2.71 ± 0.11	30.78 ± 0.23
Spent coffee ground 550 °C	76.32 ± 0.72	3.20	3.21 ± 0.04	4.11 ± 0.04	6.96 ± 0.09	6.20 ± 0.06	28.34 ± 0.15
Tea waste	46.21 ± 0.63	34.83	5.99 ± 0.07	1.82 ± 0.03	4.10 ± 0.05	7.04 ± 0.11	18.5 ± 0.20
Tea waste 250 °C	55.48 ± 0.45	30.64	5.45 ± 0.13	2.37 ± 0.01	5.31 ± 0.10	0.74 ± 0.01	21.37 ± 0.29
Tea waste 300 °C	64.24 ± 0.44	19.31	5.04 ± 0.04	2.59 ± 0.03	7.01 ± 0.07	1.80 ± 0.05	24.59 ± 0.18
Tea waste 350 °C	68.27 ± 0.53	13.38	4.43 ± 0.13	2.56 ± 0.03	8.74 ± 0.09	2.58 ± 0.12	27.22 ± 0.11
Tea waste 450 °C	72.65 ± 0.68	7.63	3.32 ± 0.13	2.49 ± 0.03	11.12 ± 0.16	2.76 ± 0.08	24.80 ± 0.29
Tea waste 550 °C	73.00 ± 0.80	3.93	2.76 ± 0.12	2.23 ± 0.01	11.75 ± 0.13	6.29 ± 0.14	25.22 ± 0.24
Walnut shell	43.53 ± 0.63	39.24	6.23 ± 0.06	0.48 ± 0.01	2.48 ± 0.06	8.05 ± 0.10	18.51 ± 0.16
Walnut shell 250 °C	55.90 ± 0.89	34.54	5.35 ± 0.11	0.52 ± 0.01	3.44 ± 0.05	0.25 ± 0.01	21.21 ± 0.26
Walnut shell 300 °C	66.88 ± 0.87	22.31	5.23 ± 0.03	0.59 ± 0.01	4.54 ± 0.02	0.45 ± 0.01	26.24 ± 0.25
Walnut shell 350 °C	74.60 ± 0.89	12.25	4.36 ± 0.09	0.69 ± 0.01	6.76 ± 0.03	1.35 ± 0.10	26.72 ± 0.19
Walnut shell 450 °C	82.06 ± 0.49	4.14	3.52 ± 0.11	0.74 ± 0.01	8.09 ± 0.05	1.47 ± 0.15	29.13 ± 0.28
Walnut shell 550 °C	83.24 ± 0.74	1.67	2.77 ± 0.11	0.82 ± 0.01	9.84 ± 0.03	1.66 ± 0.05	30.65 ± 0.28
Pistachio shell	44.53 ± 0.73	40.89	6.48 ± 0.11	0.38 ± 0.01	1.00 ± 0.03	6.71 ± 0.13	17.82 ± 0.27
Pistachio shell 250 °C	54.85 ± 0.45	37.10	5.86 ± 0.09	0.44 ± 0.01	1.46 ± 0.04	0.29 ± 0.01	21.39 ± 0.15
Pistachio shell 300 °C	66.01 ± 0.82	25.19	5.30 ± 0.13	0.49 ± 0.01	2.24 ± 0.03	0.77 ± 0.01	25.99 ± 0.18
Pistachio shell 350 °C	75.69 ± 0.93	14.94	4.42 ± 0.06	0.58 ± 0.01	2.74 ± 0.05	1.65 ± 0.08	28.43 ± 0.28
Pistachio shell 450 °C	82.37 ± 0.45	7.53	3.57 ± 0.08	0.62 ± 0.01	4.26 ± 0.09	1.65 ± 0.06	29.99 ± 0.25
Pistachio shell 550 °C	87.34 ± 0.80	2.72	2.83 ± 0.14	0.72 ± 0.01	4.66 ± 0.01	1.72 ± 0.04	31.34 ± 0.29
Peanut shell	48.97 ± 0.92	34.96	6.42 ± 0.14	1.42 ± 0.03	1.75 ± 0.02	6.48 ± 0.06	20.05 ± 0.11
Peanut shell 250 °C	56.43 ± 0.88	32.83	6.06 ± 0.05	1.56 ± 0.03	2.15 ± 0.02	0.97 ± 0.01	21.22 ± 0.24
Peanut shell 300 °C	63.36 ± 0.66	24.77	5.76 ± 0.09	1.73 ± 0.03	2.96 ± 0.09	1.43 ± 0.03	23.09 ± 0.11
Peanut shell 350 °C	72.33 ± 0.73	15.01	4.61 ± 0.15	1.94 ± 0.03	4.24 ± 0.06	1.87 ± 0.04	25.84 ± 0.23
Peanut shell 450 °C	77.12 ± 0.60	9.36	3.45 ± 0.09	1.88 ± 0.02	5.93 ± 0.10	2.26 ± 0.11	27.17 ± 0.24
Peanut shell 550 °C	81.70 ± 0.82	4.87	2.84 ± 0.10	1.96 ± 0.03	6.17 ± 0.01	2.46 ± 0.12	28.79 ± 0.27

**Table 2:** Net calorific value in the dry state of samples (MJ·kg^−1^).

	0 °C	250 °C	300 °C	350 °C	450 °C	550 °C
Spruce wood	18.6	19.02	20.83	27.31	29.48	31.56
Spent coffee ground	19.74	23.85	29.09	31.26	29.88	27.49
Tea waste	17.02	20.16	23.45	26.19	24.01	24.47
Walnut shell	16.96	20.03	25.09	25.74	28.33	30.00
Pistachio shell	16.24	20.11	24.81	27.43	29.17	30.68
Peanut shell	18.48	19.87	21.80	24.78	26.36	28.11

**Table 3:** Net calorific values of samples adjusted by material enthalpy (MJ·kg^−1^).

	0 °C	250 °C	300 °C	350 °C	450 °C	550 °C
Spruce wood	18.600	18.746	20.501	26.927	28.987	30.958
Spent coffee ground	19.740	23.576	28.761	30.877	29.387	26.888
Tea waste	17.020	19.886	23.121	25.807	23.517	23.868
Walnut shell	16.960	19.756	24.761	25.357	27.837	29.398
Pistachio shell	16.240	19.836	24.481	27.047	28.677	30.078
Peanut shell	18.480	19.596	21.471	24.397	25.867	27.508

**Table 4:** Material mass residue (%).

	0 °C	250 °C	300 °C	350 °C	450 °C	550 °C
Spruce wood	100%	83.49%	74.90%	39.56%	27.59%	21.84%
Spent coffee ground	100%	79.31%	52.63%	37.53%	22.87%	20.05%
Tea waste	100%	77.30%	54.35%	41.16%	31.83%	28.82%
Walnut shell	100%	77.49%	52.57%	36.06%	29.19%	25.81%
Pistachio shell	100%	72.35%	45.70%	31.14%	24.53%	21.98%
Peanut shell	100%	83.53%	65.04%	41.19%	31.40%	27.96%

**Table 5:** Energy residual of original mass (MJ·kg^−1^).

	0 °C	250 °C	300 °C	350 °C	450 °C	550 °C
Spruce wood	18.600	15.651	15.355	10.653	7.998	6.762
Spent coffee ground	19.740	18.697	15.136	11.587	6.720	5.392
Tea waste	17.020	15.371	12.567	10.623	7.486	6.880
Walnut shell	16.960	15.309	13.018	9.144	8.127	7.587
Pistachio shell	16.240	14.352	11.189	8.422	7.035	6.612
Peanut shell	18.480	16.369	13.965	10.049	8.123	7.691

**Table 6:** Energy residual of original mass—pellet production (MJ·kg^−1^).

	0 °C	250 °C	300 °C	350 °C	450 °C	550 °C
Spruce wood	18.492	15.561	15.275	10.610	7.969	6.738
Spent coffee ground	19.632	18.612	15.079	11.547	6.696	5.371
Tea waste	16.912	15.288	12.509	10.578	7.451	6.849
Walnut shell	16.852	15.225	12.961	9.105	8.095	7.559
Pistachio shell	16.132	14.274	11.140	8.388	7.009	6.589
Peanut shell	18.372	16.279	13.895	10.005	8.089	7.661

**Table 7:** Material price (EUR MWh^−1^).

	0 °C	250 °C	300 °C	350 °C	450 °C	550 °C
Spruce wood	20.71	24.61	**25.07**	36.10	48.06	56.84
Spent coffee ground	36.67	38.69	**47.75**	62.36	107.53	134.06
Tea waste	42.57	**47.10**	57.56	68.06	96.63	105.13
Walnut shell	42.72	47.29	**55.55**	79.07	88.94	95.25
Pistachio shell	44.63	**50.44**	64.63	85.84	102.73	109.28
Peanut shell	39.19	44.23	**51.82**	71.97	89.01	93.98

## Data Availability

The raw data supporting the conclusions of this article will be made available by the authors on request.
